# Association between IgG4 Autoantibody and Complement Abnormalities in Systemic Lupus Erythematosus

**DOI:** 10.1155/2016/2196986

**Published:** 2016-08-11

**Authors:** Qingjun Pan, Linjie Guo, Jing Wu, Jun Cai, Huanjin Liao, Qiaofen Lan, Yanxia Peng, Yiming He, Hua-feng Liu

**Affiliations:** Institute of Nephrology, Affiliated Hospital of Guangdong Medical University, Zhanjiang, Guangdong 524001, China

## Abstract

In order to investigate the association between IgG4 autoantibody and complement abnormalities in systemic lupus erythematosus (SLE), 72 newly diagnosed SLE patients, 67 rheumatoid arthritis (RA) patients, and 41 healthy normals were employed. Serum levels of antinuclear IgG4 and IgG4-specific IgM-rheumatoid factor (RF) were measured, and the correlations between serum levels of antinuclear IgG4 and several clinical parameters were analyzed. Also, the levels of IgG subclasses, C1q, and C3 deposition in lupus nephritis (LN) were detected. The results showed that serum levels of antinuclear IgG4 were higher in SLE patients relative to healthy normals (*P* < 0.01). Serum levels of antinuclear IgG4 in SLE patients were positively correlated with serum levels of total IgG4, albumin, and C3 (*r* = 0.61, *P* < 0.05; *r* = 0.40, *P* < 0.05; and *r* = 0.54, *P* < 0.05, resp.) and negatively correlated with 24-hour urinary protein (*r* = 0.49, *P* < 0.05). Serum levels of IgG4-specific IgM-RF were higher in RA patients than in SLE patients (*P* < 0.001). Also, the ratio of the deposition score for IgG4/(IgG1 + IgG2 + IgG3 + IgG4) was negatively correlated with the score for C1q and C3 deposition in LN (*r* = 0.34, *P* < 0.05; *r* = 0.51, *P* < 0.01, resp.). In summary, the IgG4 autoantibody may dampen the inflammatory response in SLE, thus maybe providing a novel therapeutic target for SLE.

## 1. Introduction

Systemic lupus erythematosus (SLE) is a systemic disease that can affect multiple organs, such as lupus nephritis (LN) resulting from autoantibody and complement-fixing immune complexes (ICs) deposition [1]. Also, complement abnormalities in SLE [2] and the function of complement molecules (C3, C1q, etc.) in the processing of ICs are well recognized [3]. However, molecular mechanisms that can affect complement consumption by specific autoantibody in SLE still need to be further investigated.

Human IgG is the main component of serum immunoglobulin, which can be divided into four subclasses: IgG1 (60–70%), IgG2 (15–20%), IgG3 (5–10%), and IgG4 (4–6%) [4, 5]. The development of IgG4-related disease (IgG4-RD) has directed the attention of autoimmune disease research to the smallest of the subclasses—IgG4. Compared with the other subclasses of IgG, the serum levels of IgG4 are low (4–8%) and IgG4 is negatively charged in the physiological environment. Additionally, the length and sequence of the amino acids in the hinge region of IgG4, located between the Fab and the C terminal of the two heavy chains (CH_2_ and CH_3_), determine the ability of IgG4 to bind to C1q and Fc*γ* receptors, and this region in IgG4 is significantly different from the hinge regions in the other IgG subclasses, resulting in a lower binding ability. Thus, IgG4 cannot stimulate the classical pathway of complement activation, even though the binding ability of IgG4 toward targeted antigen is the same as those of the other subclasses of IgG [6–9]. It can be speculated that IgG4 may have a protective effect in SLE, which may occur through the inhibition of autoantigen-mediated complement consumption.

In this study, serum levels of anti-nuclear IgG4 and IgG4-specific IgM-rheumatoid factor (IgM-RF) were detected, and the correlations between the serum levels of anti-nuclear IgG4 and several clinical parameters of SLE patients were analyzed. Additionally, the levels of IgG subclass, C1q, and C3 deposition in the kidney were detected by immunofluorescence staining to investigate the association between IgG4 autoantibody (anti-nuclear IgG4) and complement abnormalities in SLE.

## 2. Materials and Methods

### 2.1. Human Subjects

Seventy-two newly diagnosed and untreated SLE patients (10 males and 62 females with an average age of 28, ranging from 11 to 66) and 67 RA patients (11 males and 56 females with an average age of 48 ranging from 17 to 86) were selected from the Kidney Disease Department of Internal Medicine, Affiliated Hospital of Guangdong Medical University, from March 2013 to December 2014. Forty-one healthy normals (6 males and 35 females with an average age of 24, ranging from 22 to 49) were included as normal control subjects. All SLE patients were diagnosed according to the 1997 revised criteria of the American College of Rheumatology (ACR). Lupus nephritis (LN) was diagnosed in accordance with the ISN/RPS 2003 classification of LN, and rheumatoid arthritis (RA) was diagnosed in accordance with the 2009 criteria of the ACR/European League Against Rheumatism (EULAR). To eliminate the influence of IgG4-RD on IgG4 in SLE, patients who had IgG4-RD were excluded from the study. The clinical data and laboratory results of the SLE patients were collected. Written informed consent was obtained, and the protocol of this study was approved by the Medical Ethics Committee of the Affiliated Hospital of Guangdong Medical University.

### 2.2. Antibodies and Reagents

Antibodies and reagents were obtained as follows: mouse anti-human IgG4 antibody (AbD Serotec Company, UK), HRP-labeled rabbit anti-human IgG4 antibody (Boster Company, Wuhan, China), ANA Screen Test System (ZEUS ELISA*™* Kit, ZEUS Scientific, Inc., USA), Pierce*™* BCA Protein Assay Kit (Thermo Fisher Scientific Company, USA), mouse anti-human IgG1, IgG2, IgG3, and IgG4-FITC (Sigma, USA), rabbit anti-human C1q and goat anti-human C3 (MBL, Japan), and Multiskan MK3 microplate reader (Thermo Fisher Scientific Company, USA).

### 2.3. Detection of Serum Levels of IgG4 and Anti-Nuclear IgG4

Serum from patients and healthy normals was collected and stored at 80°C before testing. Serum IgG subclass levels were measured using the immunonephelometric assay [10, 11] with a molecular biology kit (Siemens, Germany, N Latex IgG4 and N Supplementary Reagent/Precipitation) and an automatic protein analyzer (Siemens BN ProSpec-I, Germany) according to the manufacturer's instructions. To measure anti-nuclear IgG4, a commercial anti-nuclear IgG ELISA Kit was modified to use anti-human IgG4-HRP instead of anti-human IgG-HRP as the secondary antibody. The Excel software was used to calculate the relative concentration of anti-nuclear IgG4 according to the optical density (OD) value measured at 450 nm.

### 2.4. Detection of Serum Levels of Albumin, C3, Scr, SUA, and 24-Hour Urinary Protein

Serum levels were measured in the clinical laboratory at our hospital as follows: albumin, Scr, and SUA were detected using an automatic biochemistry analyzer (Roche Cobas® 8000); 24-hour urinary protein was detected using an automatic biochemistry analyzer (Olympus AU2700); and serum C3 was detected by rate nephelometry using a Beckman Coulter IMMAGE 800.

### 2.5. Detection of IgG4-Specific IgM-RF in the Serum of SLE Patients by ELISA

After obtaining plasma from SLE patients, the anti-human IgG4 affinity column was manufactured using an established method [12] by conjugation of mouse anti-human IgG4 antibodies to agarose gel. The IgG4 was purified from the serum of SLE patients using the affinity column and quantified using the Pierce BCA Protein Assay Kit as per the manufacturer's instructions and then measured using a microplate reader at 570 nm. The isolated IgG4 was then coated onto plates in 0.05 M carbonate-bicarbonate coating buffer (pH 9.6) overnight at 4°C. IgG4-specific IgM-RF was then detected in the serum of SLE patients using an established ELISA method with anti-human IgM-HRP as the secondary antibody.

### 2.6. Detection of Subclasses of IgG Deposition in LN Renal Tissue by Direct Immunofluorescence Staining

One hundred forty-one cases, including 46 newly diagnosed and 65 treated SLE patients, were diagnosed with LN by renal biopsy. Kidney tissue specimens were divided and processed for immunofluorescence. Fragments from each biopsy were snap-frozen and cut using a Leica-CM1510-freezing microtome into 5 *μ*m sections, which were then stained with diluted mouse anti-human IgG1, IgG2, IgG3, IgG4, C3, or C1q-FITC using an established direct IF staining method [13–15]. Immunofluorescence sections were examined using an Olympus BX60 microscope. Immunofluorescence staining for IgG subclasses (IgG1, IgG2, IgG3, and IgG4) and for complement proteins (C3, C1q) was evaluated according to the fluorescence intensity, scored on a scale of 0–4+ as follows: 0, negative; 0.5, weak; 1, one plus; 2, two plus; 3, three plus; and 4, four plus. All slides were blindly examined by two pathologists, and ambiguous samples were reviewed to achieve a consensus.

### 2.7. Statistical Analysis

All statistical tests were performed using SPSS version 16 (SPSS, Inc., Chicago, IL, USA) and Prism 5 software (GraphPad Software, Inc., San Diego, CA, USA). All data are expressed as the mean ± standard deviation (SD). Two-group comparisons were carried out using our independent sample *t*-test. Multiple-group comparisons were performed using ANOVA, followed by the Bonferroni or Dunnett post hoc tests. The correlations between different parameters were analyzed using Spearman's rank correlation coefficient. The *P* value was considered statistically significant if it was less than 0.05.

## 3. Results

### 3.1. Serum Levels of Anti-Nuclear IgG4 in SLE Patients

**Figure 1:**
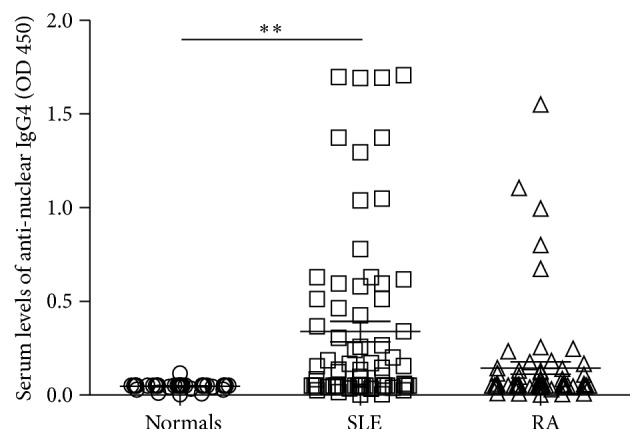
Comparison of serum levels of anti-nuclear IgG4 in various groups. Detection of serum levels of anti-nuclear IgG4 in the serum of normals and SLE and RA patients was conducted using a molecular biology kit as described in Section 2 (normal = 41, SLE = 72, and RA = 67; ^*∗∗*^ *P* < 0.01).

The serum levels of anti-nuclear IgG4 were significantly higher in SLE patients than in healthy normals (*P* < 0.01). However, there was no significant difference between the SLE patients and RA patients (*P* > 0.05) (Figure 1).

### 3.2. Correlations between Serum Levels of Anti-Nuclear IgG4 and Clinical Parameters in SLE Patients

**Figure 2:**
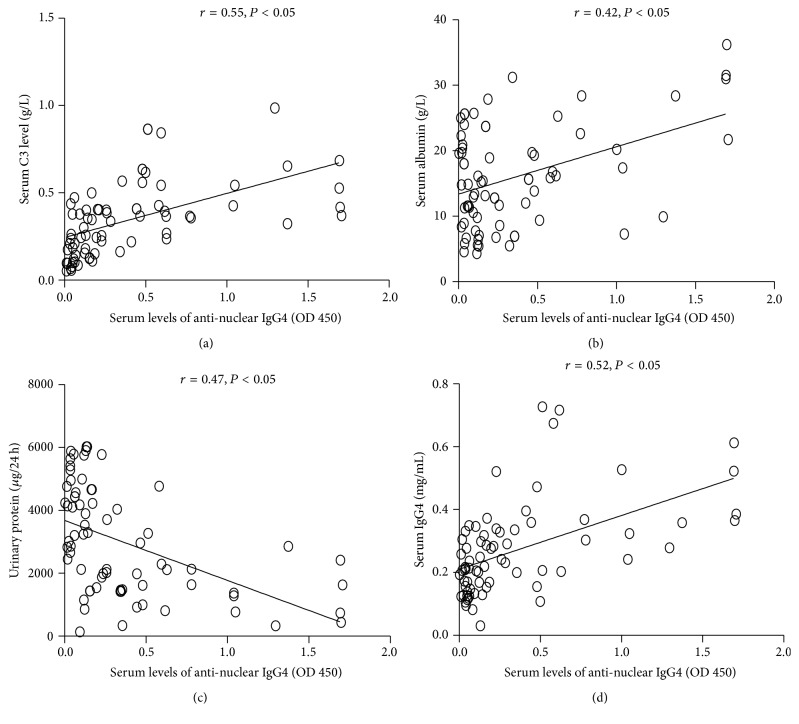
The correlations between serum levels of anti-nuclear IgG4 in SLE patients and clinical parameters. Detection of serum levels of IgG4 and anti-nuclear IgG4 was conducted by ELISA as described in Section 2. Serum levels of albumin, C3, Scr, SUA, and 24-hour urinary protein were analyzed using a commercial kit as described in Section 2.2. The correlations between serum levels of anti-nuclear IgG4 and serum levels of C3, albumin, total IgG4, and 24-hour urinary protein were analyzed using Spearman's rank correlation coefficient. The *P* value was considered statistically significant if it was less than 0.05. (*n* = 72, newly diagnosed SLE patients).

Serum levels of anti-nuclear IgG4 in SLE patients were positively correlated with serum levels of C3 and albumin (*r* = 0.55, *P* < 0.05; *r* = 0.42, *P* < 0.05) (Figures 2(a) and 2(b)), negatively associated with 24-hour urinary protein (*r* = 0.47, *P* < 0.05) (Figure 2(c)), and positively correlated with serum levels of total IgG4 (*r* = 0.52, *P* < 0.05) (Figure 2(d)). However, there was no significant difference between the serum levels of anti-nuclear IgG4 and the levels of Scr or SUA (*P* > 0.05).

### 3.3. IgG4-Specific IgM-RF in the Serum of SLE Patients

**Figure 3:**
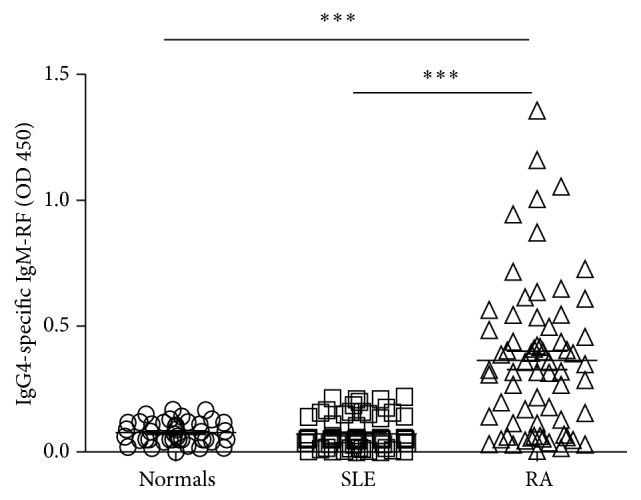
Comparison of serum levels of IgG4-specific IgM-RF in various groups. Detection of IgG4-specific IgM-RF in the serum of normals and SLE and RA patients was conducted by ELISA as described in Section 2 (normal = 41, SLE = 72, and RA = 67; ^*∗∗∗*^ *P* < 0.001).

Serum levels of IgG4-specific IgM-RF in RA patients were significantly higher than in SLE patients and healthy normals (*P* < 0.001, *P* < 0.001), but there was no significant difference between the SLE patients and the healthy normals (*P* > 0.05) (Figure 3).

### 3.4. Levels of IgG Subclass Deposition and C1q and C3 Deposition in Lupus Nephritis

**Figure 4:**
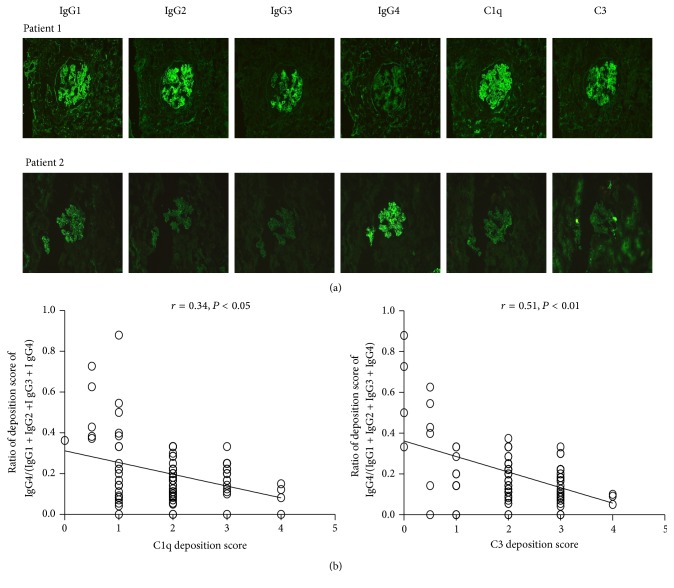
Levels of IgG subclass, C1q, and C3 deposition in lupus nephritis. (a) IgG subclass, C1q, and C3 deposition in LN renal tissue were detected by direct immunofluorescence staining as described in Section 2. (b) The correlations between the ratio of deposition score for IgG4/(IgG1 + IgG2 + IgG3 + IgG4) and the score for C1q and C3 deposition were analyzed using Spearman's rank correlation coefficient. The *P* value was considered statistically significant if it was less than 0.05 (*n* = 141, including 46 newly diagnosed LN patients and 65 LN patients treated with prednisone plus cyclophosphamide or azathioprine or hydroxychloroquine or mycophenolate mofetil).

The immunofluorescence staining results indicated that deposition of IgG subclass, C1q, and C3 was common (Figure 4(a)), and the ratio of deposition score for IgG4/(IgG1 + IgG2 + IgG3 + IgG4) was negatively correlated with the scores for C1q and C3 deposition in LN (*r* = 0.34, *P* < 0.05; *r* = 0.51, *P* < 0.01, resp.) (Figure 4(b)).

## 4. Discussion

In SLE, autoreactive B cells produce various autoantibodies against autoantigens. Anti-nuclear antibody is a disease activity marker in SLE [16] and in lupus nephritis [17], with a positivity rate of 95% to 100% at disease onset [18].

There is still controversy regarding serum levels of IgG4 in SLE, and the serum levels of anti-nuclear IgG4 have not been reported. Morland et al., Lin et al., and Sun et al. reported that serum levels of IgG4 in SLE patients had a declining trend compared to those of normal subjects [19–21]. Additionally, Kuroki et al. reported that there was no significant difference in the serum levels of IgG4 between LN patients and controls [22]. Zhang et al. reported that there was no significant difference in IgG4 or IgG4/IgG between SLE and normal subjects [23]. However, Yu reported that, compared with normal subjects, the serum levels of IgG4 were significantly higher in SLE patients who were also more likely to exhibit interstitial pneumonia and pancreatic involvement [24]; however, interstitial pneumonia and autoimmune pancreatitis have been associated with IgG4-RD, where patients show elevated serum IgG4 concentrations [25, 26]. Thus, it is unclear whether the increase in the serum levels of IgG4 in SLE patients is due to IgG4-RD. These reports suggest that there may be no significant differences in the serum levels of IgG4 when comparing SLE patients to control subjects because of variations in the detection methods or because of variability within the subject groups in this investigation (such as individual patient differences within the SLE group or SLE patients who also have LN). This suggests that the nature of IgG4 involvement in the progression of SLE remains unclear. Thus, detection of anti-nuclear IgG4, rather than total IgG4, may be a more effective clinical guideline. Our results showed that the serum levels of anti-nuclear IgG4 were significantly higher in SLE patients than in control subjects, and there were associations between the serum levels of anti-nuclear IgG4 and the clinical parameters examined. These results indicate that anti-nuclear IgG4 may play a protective role in the pathogenesis of SLE.

Because denatured IgG (including IgG4) exposes CH_2_, the Fc fragment of IgG is recognized by rheumatoid factor (RF), and the resulting formation of RF-IgG immune complexes (ICs) continues to activate complement, leading to tissue and organ damage [27]. One study indicated that approximately 26.7% of SLE patients were RF-positive, with mainly IgM-RF [28]. However, the results of our study found that the serum levels of IgG4-specific IgM-RF in SLE patients were not significantly different from those of normal subjects, though higher serum levels of IgG4-specific IgM-RF may be related to lower levels of anti-nuclear IgG4 in RA patients.

Complement abnormalities [2] and the roles of complement molecules in the processing of ICs are well recognized [3]. It is generally believed that the ICs that activate complement factors show more pathological significance, even though the induction of inflammation by ICs may be complement-dependent or complement-independent. Muso et al. found that the levels of this type of IC, which were closely associated with clinical and serological disease activities, were significantly higher in SLE patients compared to controls [29], and some experimental models have shown a role for complement in the induction of inflammatory injury [30, 31]. ICs that contain IgG4 have a limited ability to induce immune responses because of their low affinity for both Fc*γ* receptors and the C1 complement molecules [32–35]. van der Zee et al. reported that the IgG4 antibody of phospholipase A (PLA), found in the blood of beekeepers, effectively inhibited complement activation by IgG1 antibodies primarily by inhibiting the binding of C1q to IgG1 in mixed IgG1- and IgG4-containing ICs, thus reducing injury caused by immune inflammation [36]. Another study showed that IgG4 binds to the Fc portions of IgG1, IgG2, and IgG3 and blocks the Fc-mediated effector functions of IgG1 and IgG3 complexes (e.g., IgG1-class ICs, which mediate tissue damage) and may assist in the clearance of ICs by forming larger complexes that are more effectively cleared, resulting in termination of the inflammatory process [37]. Therefore, it is possible that IgG4 autoantibody (anti-nuclear IgG4) carries out an anti-inflammatory function instead of driving the disease process.

## 5. Conclusion

This study firstly investigated the association between IgG4 autoantibody (anti-nuclear IgG4) and complement abnormalities in SLE and found that the IgG4 autoantibody (anti-nuclear IgG4) may dampen the inflammatory response in SLE by competitively binding to autoantigens to form nonpathogenic ICs that result from the low affinity of IgG4 for both the Fc*γ* receptor and the C1 complement molecule, thus maybe providing a novel therapeutic target for SLE.

## Abbreviations

SLE: Systemic lupus erythematosus
RA: Rheumatoid arthritis
ELISA: Enzyme-linked immune sorbent assay
PBMC: Peripheral blood mononuclear cell
ICs: Immune complexes
IgG4-RD: IgG4-related disease
ACR: American College of Rheumatology
EULAR: European League Against Rheumatism
PLA: Phospholipase A
RF: Rheumatoid factor
LN: Lupus nephritis
Ig: Immunoglobulin.
